# Short-range correlation of stress chains near solid-to-liquid transition in active monolayers

**DOI:** 10.1098/rsif.2024.0022

**Published:** 2024-05-08

**Authors:** Siavash Monfared, Guruswami Ravichandran, José E. Andrade, Amin Doostmohammadi

**Affiliations:** ^1^ Niels Bohr Institute, University of Copenhagen, Kobenhavn, 2100, Denmark; ^2^ Division of Engineering and Applied Science, California Institute of Technology, CA, 91125, USA

**Keywords:** active matter, random percolation, stress correlation, soft matter, critical phenomena

## Abstract

Using a three-dimensional model of cell monolayers, we study the spatial organization of active *stress chains* as the monolayer transitions from a solid to a liquid state. The critical exponents that characterize this transition map the isotropic stress percolation onto the two-dimensional random percolation universality class, suggesting short-range stress correlations near this transition. This mapping is achieved via two distinct, independent pathways: (i) cell–cell adhesion and (ii) active traction forces. We unify our findings by linking the nature of this transition to high-stress fluctuations, distinctly linked to each pathway. The results elevate the importance of the transmission of mechanical information in dense active matter and provide a new context for understanding the non-equilibrium statistical physics of phase transition in active systems.

## 1. Introduction

The transition between solid and liquid states in living cells is of fundamental relevance to a range of biological processes, including cancer metastasis [1–3], wound healing [4–6] and tissue morphogenesis [7–9]. To study this transition, the concept of jamming [10,11] has been applied to inherently out-of-equilibrium living cells [12–14]. This is despite the geometrical roots of the jamming transition and its correspondence to the zero temperature and zero activity limit of a glass transition [15,16]. Notwithstanding these seminal contributions, the universality of the transition between active solid and liquid phases in cellular systems and its broader applicability are yet to be established [7,17–26].

Long-range correlations of mechanical information, i.e. force or stress, prevail in both passive glasses [27,28] and most athermal jammed granular systems [29]. In such passive systems, the long-range force or stress correlations are attributed to mechanical equilibrium [30–33]. However, in some non-equilibrium systems, long-range mechanical correlations can also emerge in two seemingly disparate systems: (i) active matter [34], where local energy injection results in the breaking of time-reversal symmetry and detailed balance [35], and (ii) boundary-driven shear of granular systems [36]. Whether a broad framework to understand these two systems exists remains to be established [37].

**Figure 1.**
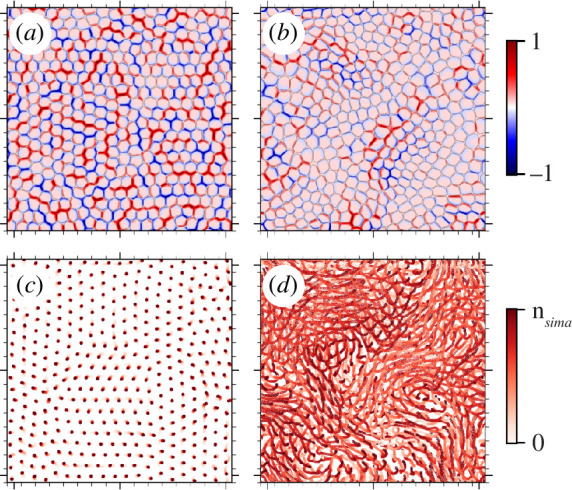
An example showing stress chains in an active monolayer. Snapshots of isotropic stress fluctuations, $\delta \sigma^{iso}=\sigma^{iso}-\langle \sigma^{iso}\rangle$ , normalized by maximum compression for (*a*) solid and (*b*) liquid states outlining both compressive (blue) and tensile (red) chains. The trajectories of individual cells during total simulation time steps ( $n_{\text{sim}}$ ) corresponding to (*c*) a solid-like state and (*d*) a liquid-like state. The colour bar indicates simulation time. (*c*) and (*d*) correspond to simulations shown in (*a*) and (*b*), respectively.

Here, using a three-dimensional model of cell monolayers, we provide evidence for the emergence of active stress chains (figure 1*a,b*) in the monolayers and show stress percolation criticality near the solid-to-liquid-like transition (figure 1*c,d*). Importantly, we establish this criticality by driving the transition via two distinct paths by independently increasing (i) cell–cell adhesion strength and (ii) active traction forces, well-established axes on the phase boundary for this transition [38,39]. Using finite-size scaling analyses, we further demonstrate that critical exponents from each path correspond to the two-dimensional random percolation universality class near the solid-to-liquid transition. Remarkably, this points to the short-range nature of stress correlations. To explain this, we provide a mechanistic basis by linking the universality class mapping to high fluctuations in stress fields with distinct signatures associated with each path. We further discuss the implications for the short-range nature of stress correlations in active monolayers and its relationship with non-equilibrium glasses, granular and biological systems.

## 2. Methods

We consider a cellular monolayer consisting of N=400 cells on a rigid substrate with its surface normal ${\vec{e}}_{n}\left(={\vec{e}}_{z}\right)={\vec{e}}_{x}\times {\vec{e}}_{y}$ and periodic boundaries in both ${\vec{e}}_{x}$ and ${\vec{e}}_{y}$ , where $\left({\vec{e}}_{x},{\vec{e}}_{y},{\vec{e}}_{z}\right)$ constitute the global orthonormal basis. Each cell i is represented by a three-dimensional phase field, $\phi_{i}=\phi_{i}\left(\vec{x},t\right)$ and initialized with radius $R_{0}$ . The phase field $\phi_{i}$ is evolved through *Model A* [40] type dynamics with an extra advective term,


(2.1) $$\partial_{t}\phi_{i}+{\vec{v}}_{i}\cdot \vec{\nabla }\phi_{i}=-\Gamma \frac{\delta F}{\delta \phi_{i}},\;i=1,\ldots ,N,$$

where ${\vec{v}}_{i}$ is the velocity of cell i , Γ represents mobility, F is the free energy functional that stabilizes cell interface and includes mechanical properties such as cell stiffness ( E ) as well as compressibility ( μ ), and puts a soft constraint on the cell volume [41–44] around $V_{0}=(4/3)\pi R_{0}^{3}$ . Additionally, the free energy comprises gradient contributions ( $\vec{\nabla }\phi$ ) that account for, and distinguish between, cell–cell ( $\omega_{cc}$ ) and cell–substrate ( $\omega_{cw}$ ) adhesions, as introduced recently [45],


(2.2) $$\begin{array}{ll} F & =\sum_{i}^{N}\frac{E}{\lambda^{2}}\int d\vec{x}\{4\phi_{i}^{2}(1-\phi_{i}{)}^{2}+\lambda^{2}(\vec{\nabla }\phi_{i}{)}^{2}\}\\ & +\sum_{i}^{N}\mu {\left(1-\frac{1}{V_{0}}\int d\vec{x}\phi_{i}^{2}\right)}^{2}+\sum_{i}^{N}\sum_{j\neq i}\frac{\kappa_{cc}}{\lambda }\int d\vec{x}\phi_{i}^{2}\phi_{j}^{2}\\ & +\sum_{i}^{N}\sum_{j\neq i}\frac{\omega_{cc}}{\lambda^{2}}\int d\vec{x}\vec{\nabla }\phi_{i}\cdot \vec{\nabla }\phi_{j}+\sum_{i}^{N}\frac{\kappa_{cw}}{\lambda }\int d\vec{x}\phi_{i}^{2}\phi_{w}^{2}\\ & +\sum_{i}^{N}\frac{\omega_{cw}}{\lambda^{2}}\int d\vec{x}\vec{\nabla }\phi_{i}\cdot \vec{\nabla }\phi_{w} \end{array},$$

where κ captures repulsion between cell–cell (subscript cc ) and cell–substrate (subscript cw ) and $\phi_{w}$ denotes a static phase field representing the substrate. Based on this free energy function, the interior and exterior of cell i corresponds to $\phi_{i}=1$ and $\phi_{i}=0$ , respectively, connected by a diffuse interface of length λ . To resolve the forces generated at the cellular interfaces, we consider an over-damped dynamics, ${\vec{T}}_{i}=\xi {\vec{v}}_{i}-{\vec{F}}_{i}^{\text{act}}=-\int d\vec{x}\left(\delta F/\delta \phi_{i}\right)\vec{\nabla }\phi_{i}$ , where ${\vec{T}}_{i}$ denotes traction [18,46,47] and contains both active and passive contributions, ξ is substrate friction and ${\vec{F}}_{i}^{\text{act}}=\alpha {\hat{F}}_{i}^{\text{pol}}$ represents an active polar force that captures the self-propulsion associated with cell polarity, constantly pushing the system out of equilibrium, with α characterizing the strength of the self-propulsion force. The dynamics of cell polarity are introduced based on contact inhibition of locomotion [48,49] by aligning the polarity of the cell to the direction of the total interaction force acting on the cell [50]. The polarization dynamics is given by $\partial_{t}\theta_{i}=-(1/\tau_{\text{pol}})|{\vec{T}}_{i}|\Delta \Theta_{i}+D_{r}\eta$ , where $\theta_{i}\in [-\pi ,\pi ]$ is the counterclockwise angle of cell polarity measured from ${\vec{e}}_{x}$ , ${\hat{F}}_{i}^{\text{pol}}=\left(\cos\theta_{i},\sin\theta_{i},0\right)$ and η is the Gaussian white noise with zero mean, unit variance, $D_{r}$ is rotational diffusivity, $\Delta \Theta_{i}$ is the angle between ${\hat{F}}_{i}^{\text{pol}}$ and ${\vec{T}}_{i}$ , with positive constant $\tau_{\text{pol}}$ setting the alignment time scale between them (see electronic supplementary material for simulation parameters). Previously, this model was tuned directly against experiments [51] and specifically chosen given its success in reproducing stress fields around nematic defects [45,47].

In passive, athermal granular systems, the jamming transition is intimately related to the packing fraction (density) of that system. However, for living cells at confluence, the collective behaviour can change from a liquid to a solid-like behaviour at a constant density. A recent study suggests density has a second-order effect on this transition [12]. Consequently, here we keep the cell density constant in our simulations, i.e. no cell proliferation nor extrusion, while ensuring confluency. For each considered pathway, we perform large-scale simulations by incrementally increasing the dimensionless cell–cell to cell–substrate adhesion ratio $\tilde{\omega }=\omega_{cc}/\omega_{cw}\in [0.1,0.5]$ for the first, and the dimensionless traction force $\tilde{\alpha }=\alpha \tau_{\text{pol}}/\xi R_{0}$ , $\tilde{\alpha }\in [0,0.8]$ , for the second pathway. We consider three distinct realizations for each case. As we show next, the considered ranges for $\tilde{\omega }$ and $\tilde{\alpha }$ capture the transition from a solid-like to a liquid state.

## 3. Results

### 3.1. Solid-to-liquid transition

**Figure 2.**
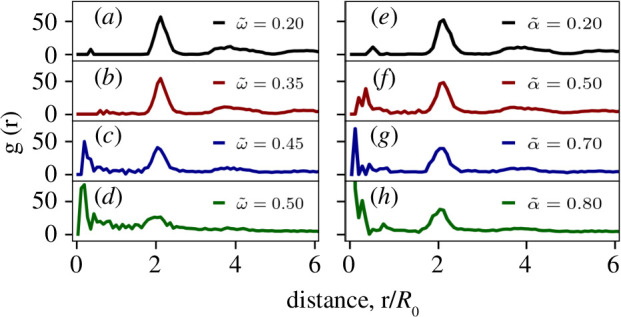
Characterization of solid-to-liquid transition via one-dimensional pair-correlation function, corresponding to time-averaged configurations, for (*a*)–(*d*) cell–cell adhesion drive and (*e*)–(*h*) active traction forces.

To characterize the solid-to-liquid-like transition, we quantify the one-dimensional pair correlation function $g\left(r\right)=(N\rho {)}^{-1}\langle \sum_{i\neq j}\delta \left(r-|{\vec{r}}_{i}-{\vec{r}}_{j}|\right)\rangle$ , where ρ denotes number density, shown in figure 2 for time-averaged configurations. For low values of $\tilde{\omega }$ and $\tilde{\alpha }$ , g(r) shows a peak near $r/R_{0}\approx 2$ , i.e. the diameter of a single cell, indicating a solid-like state. As $\tilde{\omega }$ and $\tilde{\alpha }$ increase, this peak gets smaller while another one, at a shorter distance, appears, indicative of a transition. For the time-averaged configurations, the peak $r/R_{0}\approx 0$ indicates significant overlaps between cells, relative to their initial positions, during the simulation (see figure 1*d*). For $\tilde{\omega }=0.5$ and $\tilde{\alpha }=0.8$ , the pair-correlation functions exhibit peaks at a distance much smaller than the cell diameter, suggesting a liquid-like state. Thus, the solid-to-liquid transition takes place near $\tilde{\omega }=0.45$ and $\tilde{\alpha }=0.70$ , mindful of the resolution for $\tilde{\omega }$ and $\tilde{\alpha }$ spaces. We also quantify the two-dimensional static structure factor that further confirms the identified transition points (see electronic supplementary material for details).

In the three-dimensional phase field model of active cell monolayers, *stress chains* reminiscent of force chains in passive, athermal granular systems [52,53] are observed (figure 1). In contrast with passive, athermal, convex granular systems, the stress chains in this active model exhibit both compressive and tensile stress components. This can also be the case in non-convex granular systems with the ability to entangle [54,55]. To understand the identified transition between solid–liquid states and its link to the spatial organization of mechanical information, we compute a stress field [56,57] $\sigma =\sigma (\vec{x},t)$ that encodes both active and passive contributions on a discretized domain, for node i on the complementary stress lattice,


(3.1) $$\sigma^{i}=\frac{1}{a_{0}^{3}}\sum_{j}^{N_{i}}{\vec{r}}^{ij}⊗{\vec{T}}^{j},$$

where $a_{0}^{3}$ is the volume of the unit cell, $N_{i}$ is the number of neighbours of node i on the original lattice (see electronic supplementary material for details), ${\vec{r}}^{ij}=\left({\vec{x}}^{i}-{\vec{x}}^{j}\right)$ . Given the definition of active traction for cell i , ${\vec{T}}_{i}$ , the coarse-grained stress field contains contributions from both active and passive forces.

Next, we compute the time-averaged isotropic stress field tensor, $\bar{\sigma }\left(\vec{x}\right)=(1/n_{sim})\sum_{t}^{n_{sim}}\sigma \left(\vec{x},t\right)$ and focus on the normalized isotropic stress field, ${\tilde{\bar{\sigma }}}^{iso}\left(\vec{x}\right)={\bar{\sigma }}^{iso}\left(\vec{x}\right)/{\bar{\sigma }}_{max}^{iso}$ with $n_{\text{sim}}$ number of simulation time steps, ${\bar{\sigma }}_{\text{max}}^{\text{iso}}$ the maximum value of the time-averaged isotropic stress field and ${\bar{\sigma }}^{iso}\left(\vec{x}\right)=\left(1/3\right)tr\bar{\sigma }\left(\vec{x}\right)$ , providing a measure for expansion ( $\sigma^{iso}>0$ ) and compression ( $\sigma^{iso}<0$ ) in the cell layer.

Visual inspection of the fluctuations in isotropic stress fields and the associated patterns show that for both adhesion parameter $\tilde{\omega }$ and traction force $\tilde{\alpha }$ , as the control parameter is increased and the system approaches a liquid-like state, a more disordered isotropic stress field emerges. We quantify the spatial organization of emerging stress patterns by lowering a threshold and monitoring the connectivity of the time-averaged isotropic stress field sites larger than the threshold, on a square lattice. Then, we extract the critical exponents for the stress percolation by performing finite-size scaling analyses [58,59].

We begin with quantifying the density of the spanning cluster, P(p,ℓ) , which is the probability of a site belonging to a spanning cluster as a function of the occupation probability, p , and system size, ℓ . The occupation probability, p∈[0,1] , corresponds to sites where ${\tilde{\bar{\sigma }}}^{\text{iso}}\left(\vec{x}\right)$ is greater than (1−p)×100 th percentile of the isotropic stress distribution, including both compressive and tensile stresses. In the large system size, the ‘thermodynamic limit’, i.e. ℓ→∞ , we expect the following power-law scaling near the percolation probability, $p_{c}$ , for the density of a spanning cluster P(p) characterized by the critical exponent β , $P\left(p\right)∼{\left(p-p_{c}\right)}^{\beta }$ . A cluster is a set of connected sites, and two sites are considered connected if they are nearest neighbours (eight-point connectivity). Furthermore, we quantify the average cluster size, S(p)=⟨s⟩ , where s is the size of a cluster. Near the percolation probability, the power-law scaling of S(p) quantified by critical exponent γ is $S\left(p\right)∼|p-p_{c}{|}^{-\gamma }$ and critical exponent ν , which describes the power-law scaling of correlation length ξ , i.e. average distance between two sites in the same cluster, is expected to follow $\xi ∼|p-p_{c}{|}^{-\nu }$ .

**Figure 3.**
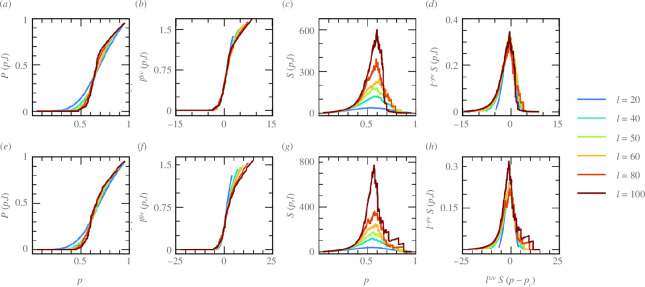
Finite-size scaling analyses of isotropic stress criticality. The density of spanning cluster P(p,ℓ) (*a, e*) and the average cluster size S(p,ℓ) (*c, g*) for different subsystems with characteristic length ℓ and their collapse after performing a finite size scaling analysis for P(p,ℓ) (*b, f*) and S(p,ℓ) (*d, h*). First row (*a–d*) corresponds to increasing dimensionless cell–cell adhesion $\tilde{\omega }=\omega_{cc}/\omega_{cw}$ and the second row (*e–h*) corresponds to the increasing strength of active traction forces $\tilde{\alpha }$ .

Both the density of the spanning cluster P(p,ℓ) and the average cluster size S(p,ℓ) are shown in figure 3 at the onset of the transition, i.e. $\tilde{\omega }$ (figure 3*a,c*) and $\tilde{\alpha }=0.7$ (figure 3*e,g*) for various system sizes, ℓ . Additionally, figure 3*b,d,f* and *h* shows the collapse of P(p,ℓ) and S(p,ℓ) when scaled with critical exponents obtained from the finite-size scaling analyses, accounting for a diverging correlation length ξ near the $p_{c}$ . Remarkably, the critical exponents corresponding to the scaling of the time-averaged isotropic stress field at the onset of the solid-to-liquid transition in the active cell layer (table 1) lead to a reasonable collapse and are in close agreement with those from the two-dimensional random percolation universality class [60]. Importantly, this agreement is obtained via two independent pathways, i.e. cell–cell adhesion and active traction force, and points to the short-range nature of stress correlations near this transition.

**Table 1.** Critical exponents obtained from finite-size scaling analyses compared with those from two-dimensional random percolation (RP).

	RP (2D)*	$\tilde{\omega }$	$\tilde{\alpha }$
$p_{c}$	0.5927	0.612±0.004	0.590±0.007
γ	2.388	2.305±0.146	2.103±0.423
β	0.1388	0.176±0.049	0.134±0.009
ν	1.333	1.422±0.051	1.243±0.198

*[60]

### 3.2. Mechanism

**Figure 4.**
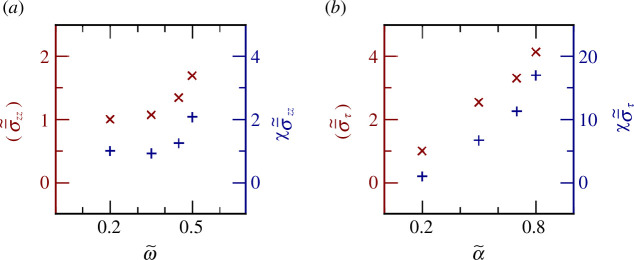
Global field statistics for time-averaged, normalized, mechanical fields. (*a*) The mean and the susceptibility of time-averaged out-of-plane stress fields for the cell–cell adhesion drive. (*b*) The mean and the susceptibility of the time-averaged maximum in-plane shear for the active traction drive. All values are normalized by those corresponding to $\tilde{\omega }=0.2$ for (*a*) and $\tilde{\alpha }=0.2$ for (*b*).

Our results thus far do not explain why short-range correlations that are characteristic of random percolation emerge in the solid-to-liquid transition of the studied active monolayers. To explore this, we analysed the stress field statistics extensively and identified two mechanical signatures associated with each path to transition. For the first path, increasing cell–cell adhesion leads to a solid-like to liquid transition. Although this counterintuitive behaviour, called the *adhesion paradox*, was observed experimentally and captured by a two-dimensional vertex model previously [61], the mechanism responsible for it is yet to be explained. Our analyses suggest increasing cell–cell adhesion results in higher global mean and field fluctuations, quantified by susceptibility, $\chi (.)=n\left(\langle .^{2}\rangle -\langle .\rangle^{2}\right)$ with n denoting field dimensions, in the out-of-plane component of the time-averaged stress tensor, ${\bar{\sigma }}_{zz}\left(\vec{x}\right)={\vec{e}}_{z}\cdot \bar{\sigma }\left(\vec{x}\right)\cdot {\vec{e}}_{z}$ . This is shown in figure 4*a* for ${\tilde{\bar{\sigma }}}_{zz}\left(\vec{x}\right)={\bar{\sigma }}_{zz}/{\bar{\sigma }}_{zz}^{max}$ . This high mean out-of-plane stress, neither accessible in two-dimensional models nor experiments, can lead to local buckling owing to cell shape changes caused by adhesion increase [62] providing a slightly more free volume needed for a transition to a liquid state [63]. Moreover, increased fluctuations are consistent with the emergence of short-range stress correlations and random percolation at the onset of the transition. For the second path to transition, increasing active polarity and hence in-plane active traction forces lead to an increase in both the global mean and field fluctuations in the maximum in-plane shear field, $\sigma_{\tau }\left(\vec{x},t\right)=(1/2)(\sigma_{max}\left(\vec{x},t\right)-\sigma_{min}\left(\vec{x},t\right))$ , where $\sigma_{max(min)}\left(\vec{x},t\right)$ is a field that corresponds to the largest (smallest) eigenvalue of the two-dimensional stress tensor (in-plane), $\sigma_{2D}\left(\vec{x},t\right)=\sigma_{xx}\left(\vec{x},t\right)({\vec{e}}_{x}⊗{\vec{e}}_{x})+\sigma_{yy}\left(\vec{x},t\right)({\vec{e}}_{y}⊗{\vec{e}}_{y})+\sigma_{xy}\left(\vec{x},t\right)({\vec{e}}_{x}⊗{\vec{e}}_{y}+{\vec{e}}_{y}⊗{\vec{e}}_{x})$ . This is shown in figure 4*b* for the time-averaged field, ${\bar{\sigma }}_{\tau }\left(\vec{x}\right)$ , normalized by its maximum, ${\bar{\sigma }}_{\tau }^{max}$ , ${\tilde{\bar{\sigma }}}_{\tau }\left(\vec{x}\right)={\bar{\sigma }}_{\tau }/{\bar{\sigma }}_{\tau }^{max}$ . Thus, the transition driven by increasing active traction leads to an increase in both mean and fluctuations of local, maximum shear, from which emerges stress percolation, near the transition. We note that an important observation here is the encoding of different types of mechanical information in the isotropic stress field. In this vein, our approach has the potential to unify disparate observations in biological systems, such as percolations based on (i) cell connectivity [25] and (ii) edge tension network [64,65] through understanding the transmission of mechanical information.

## 4. Discussion

The critical exponents obtained by finite-size scaling analyses of time-averaged isotropic stress fields map the solid-to-liquid transition in active monolayers to a two-dimensional random percolation universality class. Thus, we reveal the short-range nature of stress correlations near this transition, attuned to differing critical exponents for percolation in systems with long-range correlated disorder [66]. In this vein, our findings lend further support to the viewpoint that in passive amorphous solids, it is the mechanical equilibrium condition that gives rise to long-range stress correlations [30–33]. Importantly, we establish the criticality of the active solid-to-liquid transition via two distinct paths, which are both well established experimentally [23,61].

A particularly appealing feature of our approach is the implicit manifestation of physical interactions as mechanical information in isotropic stress fields, given the two distinct drives considered in our study. This is important since direct measurement of traction forces and mechanical stress fields is experimentally accessible [46,67] and could be used to confirm the proposed critical behaviour. Our results are particularly illuminating since recent experiments highlight the dominant role of traction forces in deriving the solid-to-liquid transition in epithelial tissues [23]. Activity in our study is owing to active polarity. The particular model we use is tuned directly by experiments [51] and specifically chosen given its success in representing cell motility by reproducing stress patterns around nematic defects [45,47]. In this vein, a recent study suggests that the active glassy dynamics are mute to the details of such a model [68].

The critical exponents were estimated by extracting squared subsystems of characteristic length ℓ∈[20,100] , spanning almost a decade of length scales, with dimensions $(\ell +1{)}^{2}$ given the lattice spacing $a_{0}=1\ll R_{0}$ , from the time-averaged isotropic stress fields with total dimensions of $(L_{x}+1)\times (L_{y}+1)=$ 103 041 (see electronic supplementary material for finite-size scaling analyses). While our analyses can definitely benefit from more statistics, we are confident in its robustness since we obtain similar critical exponents from two distinct and independent paths towards solid-to-liquid transition. Additionally, we used site percolation on a square lattice, given the nature of our simulations, but it is well known that critical exponents for random percolation universality class are robust to model details [60,66,69], e.g. bond versus site percolation and lattice type. Thus, we do not expect our results to change based on lattice types, e.g. triangular, excluding the Bethe lattice.

The finite-size scaling analyses were performed on two-dimensional isotropic stress fields that embed the out-of-plane stress component $\sigma_{zz}$ . Furthermore, the three-dimensional nature of our modelling approach presents other unique advantages including the ability to tune independently and explicitly for cell–cell and cell–substrate interactions. Most importantly, we can ensure that, within the range of studied parameters, the solid-to-liquid transition is not owing to an extrusion event, altering the confluent state. This cannot be done with two-dimensional modelling approaches.

We also examined potential links to jamming transition in passive granular systems [70,71]. Interestingly, a recent study suggests short-range force correlation in jammed granular systems [72], in agreement with our findings. However, this contrasts with other studies that point to long-range correlations in such systems [29,53]. This may be owing to the dependence of critical parameters associated with jamming transition on the nature of the global load, e.g. shear versus isotropic compression, as well as the loading rate [73,74]. Moreover, our results differ from the critical exponents associated with standard rigidity percolation [75,76], indicating distinct universality classes. In this vein, efforts to link the jamming transition to rigidity percolation remain elusive [71,77]. Additionally, contact connectivity percolation [78,79] has critical exponents that are different from those for random percolation [80,81]. Our results also contrast with non-equilibrium, boundary-driven sheared granular systems exhibiting long-range force correlation [36] and delineate the difference between an active matter system, driven by local injection of energy and boundary-driven non-equilibrium systems. In this vein, our study provides a new context to explore potential links between dense active matter and glasses [82–84]. Exploring other pathways to transition, such as intercellular friction [85] and the role of mechanochemical waves [86], are exciting avenues for near-future studies. Moreover, progress is made towards direct measurement of traction forces and mechanical stress fields in three dimensions [87] which can be used to probe the proposed universality in cellular monolayers in experiments. Furthermore, understanding the spatial organization of stresses and their correlations in active p-atic liquid crystal models beyond onefold (p=1) rotational symmetry, i.e. active polarity, and coupling with active poroelasticity [88] are the natural next steps for this study.

## Data Availability

The current manuscript is a computational study, so no data have been generated for this manuscript. Modelling code is available here [89]. Data and post-processing codes can be found here [90]. Electronic supplementary material is available online at [91].

## References

[1] Oswald L , Grosser S , Smith DM , Käs JA . 2017 Jamming transitions in cancer. J. Phys. D Appl. Phys. **50** , 483001. (10.1088/1361-6463/aa8e83) 29628530 PMC5884432

[2] Grosser S , et al . 2021 Cell and nucleus shape as an indicator of tissue fluidity in carcinoma. Phys. Rev. X **11** , 011033. (10.1103/PhysRevX.11.011033)

[3] Blauth E , Kubitschke H , Gottheil P , Grosser S , Käs JA . 2021 Jamming in embryogenesis and cancer progression. Front. Phys. **9** . (10.3389/fphy.2021.666709)

[4] Kim JH *et al* . 2013 Propulsion and navigation within the advancing monolayer sheet. Nat. Mater. **12** , 856–863. (10.1038/nmat3689) 23793160 PMC3750079

[5] Tetley RJ , Staddon MF , Heller D , Hoppe A , Banerjee S , Mao Y . 2019 Tissue fluidity promotes epithelial wound healing. Nat. Phys. **15** , 1195–1203. (10.1038/s41567-019-0618-1) 31700525 PMC6837871

[6] Jain A *et al* . 2020 Regionalized tissue fluidization is required for epithelial gap closure during insect gastrulation. Nat. Commun. **11** , 5604. (10.1038/s41467-020-19356-x) 33154375 PMC7645651

[7] Mongera A *et al* . 2018 A fluid-to-solid jamming transition underlies vertebrate body axis elongation. Nature **561** , 401–405. (10.1038/s41586-018-0479-2) 30185907 PMC6148385

[8] Petridou NI , Grigolon S , Salbreux G , *et al* . 2019 Fluidization-mediated tissue spreading by mitotic cell rounding and non-canonical Wnt signalling. Nat. Cell Biol. **21** , 169–178. (10.1038/s41556-018-0247-4) 30559456

[9] Petridou NI , Heisenberg CP . 2019 Tissue rheology in embryonic organization. EMBO J. **38** , e102497. (10.15252/embj.2019102497) 31512749 PMC6792012

[10] Liu AJ , Nagel SR . 1998 Jamming is not just cool any more. Nature **396** , 21–22. (10.1038/23819)

[11] Cates ME , Wittmer JP , Bouchaud JP , Claudin P . 1998 Jamming, force chains, and fragile matter. Phys. Rev. Lett. **81** , 1841–1844. (10.1103/PhysRevLett.81.1841)

[12] Garcia S , Hannezo E , Elgeti J . 2015 Physics of active jamming during collective cellular motion in a monolayer. Proc. Natl Acad. Sci. USA **112** , 15 314–15 319. (10.1073/pnas.1510973112) PMC4687586 26627719

[13] Bi D , Lopez JH , Schwarz JM , Manning ML . 2015 A density-independent rigidity transition in biological tissues. Nat. Phys. **11** , 1074–1079. (10.1038/nphys3471)

[14] Atia L *et al* . 2018 Geometric constraints during epithelial jamming. Nat. Phys. **14** , 613–620. (10.1038/s41567-018-0089-9) 30151030 PMC6108541

[15] Parisi G , Zamponi F . 2010 Mean-field theory of hard sphere glasses and jamming. Rev. Mod. Phys. **82** , 789–845. (10.1103/RevModPhys.82.789)

[16] Berthier L , Flenner E , Szamel G . 2019 Glassy dynamics in dense systems of active particles. J. Chem. Phys. **150** , 200901. (10.1063/1.5093240) 31153189

[17] Farhadifar R , Röper JC , Aigouy B . 2007 The influence of cell mechanics, cell-cell interactions, and proliferation on epithelial packing. Curr. Biol. **17** , 2095–2104. (10.1016/j.cub.2007.11.049) 18082406

[18] Trepat X , Wasserman MR , Angelini TE , Millet E , Weitz DA , Butler JP , Fredberg JJ . 2009 Physical forces during collective cell migration. Nat. Phys. **5** , 426–430. (10.1038/nphys1269)

[19] Angelini TE , Hannezo E , Trepat X , Fredberg JJ , Weitz DA . 2010 Cell migration driven by cooperative substrate deformation patterns. Phys. Rev. Lett. **104** , 168104. (10.1103/PhysRevLett.104.168104) 20482085 PMC3947506

[20] Nnetu KD , Knorr M , Käs J , Zink M . 2012 The impact of jamming on boundaries of collectively moving weak-interacting cells. New J. Phys. **14** , 115012. (10.1088/1367-2630/14/11/115012)

[21] Sussman DM , Merkel M . 2018 No unjamming transition in a Voronoi model of biological tissue. Soft Matter **14** , 3397–3403. (10.1039/c7sm02127e) 29667689

[22] Czajkowski M , Sussman DM , Marchetti MC , Manning ML . 2019 Glassy dynamics in models of confluent tissue with mitosis and apoptosis. Soft Matter **15** , 9133–9149. (10.1039/c9sm00916g) 31674622

[23] Saraswathibhatla A , Notbohm J . 2020 Tractions and stress fibers control cell shape and rearrangements in collective cell migration. Phys. Rev. X **10** . (10.1103/PhysRevX.10.011016)

[24] Kim S , Pochitaloff M , Stooke-Vaughan GA , Campàs O . 2021 Embryonic tissues as active foams. Nat. Phys. **17** , 859–866. (10.1038/s41567-021-01215-1) 34367313 PMC8336761

[25] Petridou NI , Corominas-Murtra B , Heisenberg CP , Hannezo E . 2021 Rigidity percolation uncovers a structural basis for embryonic tissue phase transitions. Cell **184** , 1914–1928. (10.1016/j.cell.2021.02.017) 33730596 PMC8055543

[26] Hopkins A , Chiang M , Loewe B , Marenduzzo D , Marchetti MC . 2022 Local yield and compliance in active cell monolayers. Phys. Rev. Lett. **129** , 148101. (10.1103/PhysRevLett.129.148101) 36240394

[27] Maier M Zippelius A Fuchs M . 2017 Emergence of long-ranged stress correlations at the liquid to glass transition. Phys. Rev. Lett. **119** , 265701. (10.1103/PhysRevLett.119.265701) 29328698

[28] Szamel G Flenner E . 2011 Emergence of long-range correlations and rigidity at the dynamic glass transition. Phys. Rev. Lett. **107** , 105505. (10.1103/PhysRevLett.107.105505) 21981512

[29] Ostojic S , Somfai E , Nienhuis B . 2006 Scale invariance and universality of force networks in static granular matter. Nature **439** , 828–830. (10.1038/nature04549) 16482153

[30] Henkes S , Chakraborty B . 2009 Statistical mechanics framework for static granular matter. Phys. Rev. E. Stat. Nonlin. Soft Matter Phys. **79** , 061301. (10.1103/PhysRevE.79.061301) 19658495

[31] Lemaître A . 2018 Stress correlations in glasses. J. Chem. Phys. **149** , 104107. (10.1063/1.5041461) 30219008

[32] DeGiuli E . 2018 Field theory for amorphous solids. Phys. Rev. Lett. **121** , 118001. (10.1103/PhysRevLett.121.118001) 30265104

[33] Tong H , Sengupta S , Tanaka H . 2020 Emergent solidity of amorphous materials as a consequence of mechanical self-organisation. Nat. Commun. **11** , 4863. (10.1038/s41467-020-18663-7) 32978393 PMC7519136

[34] Henkes S , Kostanjevec K , Collinson JM . 2020 Dense active matter model of motion patterns in confluent cell monolayers. Nat. Commun. **11** , 1405. (10.1038/s41467-020-15164-5) 32179745 PMC7075903

[35] Bowick MJ , Fakhri N , Marchetti MC , Ramaswamy S . 2022 Symmetry, thermodynamics, and topology in active matter. Phys. Rev. X **12** , 010501. (10.1103/PhysRevX.12.010501)

[36] Dashti H , Saberi AA , Rahbari SHE , Kurths J . 2023 Emergence of rigidity percolation in flowing granular systems. Sci. Adv **9** , eadh5586. (10.1126/sciadv.adh5586) 37656797 PMC12488052

[37] Morse PK , Roy S , Agoritsas E . 2021 A direct link between active matter and sheared granular systems. Proc. Natl Acad. Sci. USA **118** , e2019909118. (10.1073/pnas.2019909118) 33931504 PMC8106327

[38] Sadati M , Taheri Qazvini N , Krishnan R . 2013 Collective migration and cell jamming. Differentiation **86** , 121–125. (10.1016/j.diff.2013.02.005) 23791490 PMC3795803

[39] Bi D , Yang X , Marchetti MC , Manning ML . 2016 Motility-driven glass and jamming transitions in biological tissues. Phys. Rev. X **6** , 021011. (10.1103/PhysRevX.6.021011) 28966874 PMC5619672

[40] Hohenberg PC , Halperin BI . 1977 Theory of dynamic critical phenomena. Rev. Mod. Phys. **49** , 435–479. (10.1103/RevModPhys.49.435)

[41] Palmieri B , Bresler Y , Wirtz D , Grant M . 2015 Multiple scale model for cell migration in monolayers: elastic mismatch between cells enhances motility. Sci. Rep. **5** , 11745. (10.1038/srep11745) 26134134 PMC5155609

[42] Aranson IS . 2015 Physical models of cell motility: biological and medical physics, biomedical engineering, 1st edn. Basel, Switzerland: Springer International Publishing. (10.1007/978-3-319-24448-8)

[43] Camley BA , Rappel WJ . 2017 Physical models of collective cell motility: from cell to tissue. J. Phys. D Appl. Phys. **50** , 113002. (10.1088/1361-6463/aa56fe) 28989187 PMC5625300

[44] Mueller R , Yeomans JM , Doostmohammadi A . 2019 Emergence of active nematic behavior in monolayers of isotropic cells. Phys. Rev. Lett. **122** , 048004. (10.1103/PhysRevLett.122.048004) 30768306

[45] Monfared S , Ravichandran G , Andrade J , Doostmohammadi A . 2023 Mechanical basis and topological routes to cell elimination. Elife **12** , e82435. (10.7554/eLife.82435) 37070647 PMC10112887

[46] Brugués A *et al* . 2014 Forces driving epithelial wound healing. Nat. Phys. **10** , 683–690. (10.1038/nphys3040) 27340423 PMC4915550

[47] Saw TB *et al* . 2017 Topological defects in epithelia govern cell death and extrusion. Nature **544** , 212–216. (10.1038/nature21718) 28406198 PMC5439518

[48] Abercrombie M , Heaysman JEM . 1954 Observations on the social behaviour of cells in tissue culture. Exp. Cell Res. **6** , 293–306. (10.1016/0014-4827(54)90176-7) 13173482

[49] Abercrombie M . 1979 Contact inhibition and malignancy. Nature **281** , 259–262. (10.1038/281259a0) 551275

[50] Smeets B , Alert R , Pešek J . 2016 Emergent structures and dynamics of cell colonies by contact inhibition of locomotion. Proc. Natl Acad. Sci. USA **113** , 14 621–14 626. (10.1073/pnas.1521151113) 27930287 PMC5187738

[51] Peyret G , Mueller R , d’Alessandro J , *et al* . 2019 Sustained oscillations of epithelial cell sheets. Biophys. J. **117** , 464–478. (10.1016/j.bpj.2019.06.013) 31307676 PMC6697349

[52] Liu CH , Nagel SR , Schecter DA , *et al* . 1995 Force fluctuations in bead packs. Science **269** , 513–515. (10.1126/science.269.5223.513) 17842361

[53] Majmudar TS , Behringer RP . 2005 Contact force measurements and stress-induced anisotropy in granular materials. Nature **435** , 1079–1082. (10.1038/nature03805) 15973358

[54] de Macedo R , Monfared S , Karapiperis K , Andrade JE . 2023 What is shape? Characterizing particle morphology with genetic algorithms and deep generative models. Granular Matter **25** , 2. (10.1007/s10035-022-01282-y)

[55] Karapiperis K , Monfared S , Macedo R de , Richardson S , Andrade JE . 2022 Stress transmission in entangled granular structures. Granular Matter **24** . (10.1007/s10035-022-01252-4)

[56] Irving JH , Kirkwood JG . 1950 The statistical mechanical theory of transport processes. IV. The equations of hydrodynamics. J. Chem. Phys. **18** , 817–829. (10.1063/1.1747782)

[57] Christoffersen J , Mehrabadi MM , Nemat-Nasser S . 1981 A micromechanical description of granular material behavior. J. Appl. Mech. **48** , 339–344. (10.1115/1.3157619)

[58] Fisher ME Ferdinand AE . 1967 Interfacial, boundary, and size effects at critical points. Phys. Rev. Lett. **19** , 169–172. (10.1103/PhysRevLett.19.169)

[59] Fisher ME , Barber MN . 1972 Scaling theory for finite-size effects in the critical region. Phys. Rev. Lett. **28** , 1516–1519. (10.1103/PhysRevLett.28.1516)

[60] Stauffer D , Aharony A . 2018 Introduction to percolation theory. London, UK: Taylor & Francis.

[61] Park JA *et al* . 2015 Unjamming and cell shape in the asthmatic airway epithelium. Nat. Mater. **14** , 1040–1048. (10.1038/nmat4357) 26237129 PMC4666305

[62] Hannezo E , Prost J , Joanny JF . 2014 Theory of epithelial sheet morphology in three dimensions. Proc. Natl Acad. Sci. USA **111** , 27–32. (10.1073/pnas.1312076111) 24367079 PMC3890844

[63] Cohen MH , Turnbull D . 1959 Molecular transport in liquids and glasses. J. Chem. Phys. **31** , 1164–1169. (10.1063/1.1730566)

[64] Li X , Das A , Bi D . 2019 Mechanical heterogeneity in tissues promotes rigidity and controls cellular invasion. Phys. Rev. Lett. **123** . (10.1103/PhysRevLett.123.058101) 31491312

[65] Fuhs T *et al* . 2022 Rigid tumours contain soft cancer cells. Nat. Phys. **18** , 1510–1519. (10.1038/s41567-022-01755-0)

[66] Schrenk KJ , Posé N , Kranz JJ , van Kessenich LVM , Araújo NAM , Herrmann HJ . 2013 Percolation with long-range correlated disorder. Phys. Rev. E. Stat. Nonlin. Soft Matter Phys. **88** , 052102. (10.1103/PhysRevE.88.052102) 24329209

[67] Ladoux B . 2009 Cells guided on their journey. Nat. Phys. **5** , 377–378. (10.1038/nphys1281)

[68] Debets VE , Janssen LMC . 2022 Active glassy dynamics is unaffected by the microscopic details of self-propulsion. J. Chem. Phys. **157** , 224902. (10.1063/5.0127569) 36546821

[69] Grimmett GR . 2010 Percolation. Berlin, Germany: Springer. (Grundlehren der mathematischen Wissenschaften).

[70] O’Hern CS , Langer SA , Liu AJ , Nagel SR . 2002 Random packings of frictionless particles. Phys. Rev. Lett. **88** , 075507. (10.1103/PhysRevLett.88.075507) 11863912

[71] O’Hern CS , Silbert LE , Liu AJ , Nagel SR . 2003 Jamming at zero temperature and zero applied stress: the epitome of disorder. Phys. Rev. E Stat. Nonlin. Soft Matter Phys. **68** , 011306. (10.1103/PhysRevE.68.011306) 12935136

[72] Pathak SN , Esposito V , Coniglio A , Ciamarra MP . 2017 Force percolation transition of jammed granular systems. Phys. Rev. E **96** , 042901. (10.1103/PhysRevE.96.042901) 29347617

[73] Peshkov A , Teitel S . 2021 Critical scaling of compression-driven jamming of athermal frictionless spheres in suspension. Phys. Rev. E **103** , L040901. (10.1103/PhysRevE.103.L040901) 34006006

[74] Jin Y , Yoshino H . 2021 A jamming plane of sphere packings. Proc. Natl Acad. Sci. **118** , 14. (10.1073/pnas.2021794118) PMC8040821 33795514

[75] Jacobs DJ , Thorpe MF . 1996 Generic rigidity percolation in two dimensions. Phys. Rev. E. **53** , 3682–3693. (10.1103/physreve.53.3682) 9964678

[76] Moukarzel C , Duxbury PM . 1999 Comparison of rigidity and connectivity percolation in two dimensions. Phys. Rev. E **59** , 2614–2622. (10.1103/PhysRevE.59.2614)

[77] Ellenbroek WG , Hagh VF , Kumar A , Thorpe MF , van Hecke M . 2015 Rigidity loss in disordered systems: three scenarios. Phys. Rev. Lett. **114** , 135501. (10.1103/PhysRevLett.114.135501) 25884127

[78] Aharonov E , Sparks D . 1999 Rigidity phase transition in granular packings. Phys. Rev. E. **60** , 6890–6896. (10.1103/physreve.60.6890) 11970627

[79] Arévalo R , Zuriguel I , Maza D . 2010 Topology of the force network in the jamming transition of an isotropically compressed granular packing. Phys. Rev. E. **81** , 041302. (10.1103/PhysRevE.81.041302) 20481712

[80] Gawlinski ET , Stanley HE . 1981 Continuum percolation in two dimensions: Monte Carlo tests of scaling and universality for non-interacting discs. J. Phys. A Math. Gen. **14** , L291–L299. (10.1088/0305-4470/14/8/007)

[81] Shen T , O’Hern CS , Shattuck MD . 2012 Contact percolation transition in athermal particulate systems. Phys. Rev. E. **85** , 011308. (10.1103/PhysRevE.85.011308) 22400566

[82] Berthier L , Kurchan J . 2013 Non-equilibrium glass transitions in driven and active matter. Nat. Phys. **9** , 310–314. (10.1038/nphys2592)

[83] Fodor É , Nardini C , Cates ME . 2016 How far from equilibrium is active matter? Phys. Rev. Lett. **117** , 038103. (10.1103/PhysRevLett.117.038103) 27472145

[84] Morse PK , Roy S , Agoritsas E . 2021 A direct link between active matter and sheared granular systems. Proc. Natl Acad. Sci. USA **118** , e2019909118. (10.1073/pnas.2019909118) 33931504 PMC8106327

[85] Chiang M , Hopkins A , Benjamin Loewe MCM , Marenduzzo D . 2023 Intercellular friction and motility drive orientational order in cell monolayers. arXiv. (10.48550/arXiv.2310.20465) PMC11459176 39302997

[86] Boocock D , Hirashima T , Hannezo E . 2023 Interplay between mechanochemical patterning and glassy dynamics in cellular monolayers. PRX Life **1** . (10.1103/PRXLife.1.013001)

[87] Cheung BCH , Abbed RJ , Wu M , Leggett SE . 2024 3D traction force microscopy in biological gels: from single cells to multicellular spheroids. Annu. Rev. Biomed. Eng. **26** . (10.1146/annurev-bioeng-103122-031130) PMC12914736 38316064

[88] Munjal A , Hannezo E , Tsai TYC , Mitchison TJ , Megason SG . 2021 Extracellular hyaluronate pressure shaped by cellular tethers drives tissue morphogenesis. Cell **184** , 6313–6325. (10.1016/j.cell.2021.11.025) 34942099 PMC8722442

[89] Siavashmonfared . 2024 Data from: Siavashmonfared/Celadro_Three_Dimensional: 3D_Active_Liquid_Droplets (V.2021.08). Zenodo. (10.5281/zenodo.10790897)

[90] Khosh Sokhan Monfared S , Ravichandran G , Andrade J , Doostmohammadi A . 2024 Short-range correlation of stress chains near solid-to-liquid transition in active monolayers [Dataset]. Dryad. (10.5061/dryad.18931zd4k) PMC11077009 38715321

[91] Monfared S , Ravichandran G , Andrade J , Doostmohammadi A . 2024 Supplementary material from: Short-range correlation of stress chains near solid-to-liquid transition in active monolayers. FigShare (10.6084/m9.figshare.c.7209354) PMC11077009 38715321

